# The Link Between Preterm Birth and Long-Term Renal Consequences: Current Knowledge and Emerging Therapeutic Targets

**DOI:** 10.3390/biomedicines14030517

**Published:** 2026-02-26

**Authors:** John Dotis, Alexandra Skarlatou, Maria Fourikou, Athina Papadopoulou, Elpis Chochliourou

**Affiliations:** 1 Third Department of Pediatrics, Aristotle University of Thessaloniki, Hippokration Hospital, 54642 Thessaloniki, Greece; mfour@auth.gr (M.F.); echochl@auth.gr (E.C.); 2 Neonatal Intensive Care Unit, Pediatric Department, Democritus University of Thrace, University General Hospital of Alexandroupolis, 68100 Alexandroupolis, Greece; alskarlatou@pgna.gr; 3 First Department of Pediatrics, Aristotle University of Thessaloniki, Hippokration Hospital, 54642 Thessaloniki, Greece; athinapap80@yahoo.gr

**Keywords:** preterm birth, nephron endowment, chronic kidney disease, neonatal acute kidney injury, hyperfiltration, biomarkers, hypertension, long-term follow-up

## Abstract

**Background/Objectives:** Preterm birth interrupts nephrogenesis during a critical developmental window, resulting in reduced nephron endowment and lifelong renal vulnerability. Evidence indicates that individuals born preterm are at increased risk for hypertension, albuminuria, and chronic kidney disease (CKD) across the life course. This review synthesizes current evidence linking prematurity with adverse renal outcomes, explores key pathophysiological mechanisms, and discusses emerging biomarkers together with therapeutic strategies. **Methods:** This comprehensive review integrates evidence from clinical cohort studies, population-based registries, meta-analyses and experimental models. Factors such as neonatal acute kidney injury (AKI), nephrotoxic exposures and cardiometabolic interactions were integrated to provide a life-course perspective. **Results:** Preterm birth leads to reduced nephron endowment, compensatory glomerular hypertrophy, and hyperfiltration, which predispose to progressive nephron loss. Postnatal factors, including neonatal AKI, inflammation, nephrotoxic medications, and later cardiometabolic stress, act as cumulative “hits”, accelerating renal injury trajectories. Clinical studies demonstrate a higher prevalence of reduced estimated glomerular filtration rate, albuminuria, elevated blood pressure, and smaller kidney volumes from childhood into adulthood. Emerging biomarkers such as cystatin C, alongside imaging-based estimates of nephron endowment, may enhance early risk stratification. **Conclusions:** Preterm birth represents an independent, lifelong risk factor for CKD through combined developmental and postnatal mechanisms. Structured long-term surveillance and early preventive strategies are essential to preserve renal reserve in this population. Advances in biomarker-guided monitoring and targeted interventions may enable earlier identification of high-risk individuals and support precision approaches to nephroprotection after prematurity.

## 1. Introduction

Preterm birth, defined as delivery before 37 completed weeks of gestation, remains a major global public health challenge, affecting approximately 10–11% of live births worldwide. Although advances in neonatal intensive care have markedly improved survival rates, including among extremely preterm infants, accumulating evidence indicates that improved survival is accompanied by increased long-term morbidity across multiple organ systems, including the kidney [1].

Renal vulnerability after preterm birth reflects the timing of human nephrogenesis, which normally continues until approximately 34–36 weeks’ gestation. Preterm delivery therefore interrupts active nephron formation, resulting in reduced nephron endowment at birth [2]. Structural and experimental studies consistently demonstrate fewer nephrons, altered glomerular architecture, and compensatory enlargement of remaining glomeruli in individuals born preterm, changes that may persist into later life [3]. Reduced nephron endowment increases functional demand on the remaining filtering units, initiating adaptive hemodynamic responses that may predispose to progressive nephron loss over time [4]. This framework links developmental nephron deficit to the higher prevalence of hypertension, albuminuria, and diminished renal reserve observed in individuals born preterm [5].

Large cohort studies demonstrate that preterm birth is independently associated with lower estimated glomerular filtration rate (eGFR), higher blood pressure, and increased risk of chronic kidney disease (CKD) from childhood into mid-adulthood, even after adjustment for socioeconomic and perinatal confounders [1,6]. Importantly, risk increases with decreasing gestational age, supporting a graded relationship between developmental immaturity and later renal vulnerability.

Beyond structural nephron deficit, postnatal exposures may further shape renal trajectories. Episodes of neonatal acute kidney injury (AKI), exposure to nephrotoxic medications, altered renal perfusion, oxidative stress, and systemic inflammation may interact with developmental susceptibility to accelerate injury pathways [7]. Rapid postnatal catch-up growth and later cardiometabolic stress may act as additional modifiers, imposing increased functional demand on a developmentally constrained nephron pool [8].

Collectively, these observations align with the developmental origins of health and disease (DOHaD) framework, whereby early-life disturbances in organ development exert lasting structural and functional consequences. Within this context, prematurity should be recognized as a developmental determinant of CKD risk, with implications for structured long-term surveillance and preventive strategies.

The aim of this comprehensive review is to synthesize current clinical and experimental evidence linking preterm birth to long-term renal outcomes, to delineate the principal pathophysiological mechanisms involved, and to discuss emerging biomarkers, preventive strategies, and potential therapeutic targets. A clearer understanding of these pathways is essential to reduce the future burden of CKD in the growing population of individuals born prematurely.

## 2. Kidney Development and the Impact of Preterm Birth

### 2.1. Normal Nephrogenesis: Timing and Vulnerable Windows

Human nephrogenesis is a tightly regulated developmental process that occurs predominantly during the third trimester and is typically completed between 34 and 36 weeks’ gestation. Beyond this period, no new nephrons are formed, rendering nephron endowment a fixed biological parameter throughout life [2]. Final nephron formation requires coordinated ureteric bud branching, mesenchymal differentiation, and vascular integration—processes highly sensitive to environmental perturbations [9].

This late gestational window represents a critical period of vulnerability. Disturbances in oxygenation, growth factor signaling, and metabolic supply may permanently alter renal structure, with experimental and human data linking such disruptions to reduced nephron number and altered glomerular architecture [8]. Preterm birth therefore interrupts nephrogenesis during active maturation, abruptly shifting renal development from a controlled intrauterine environment to a physiologically stressful extrauterine setting [4].

The neonatal intensive care environment further exposes immature kidneys to fluctuating oxygen levels, systemic inflammation, hemodynamic instability, and pharmacologic interventions, all of which may interfere with ongoing renal maturation [6]. Because nephron progenitor cells remain active during this period, these exposures may compromise both nephron quantity and structural integrity [10]. Prematurity should thus be conceptualized as a developmental disruption with potential lifelong renal consequences.

### 2.2. Incomplete Nephrogenesis and Reduced Nephron Endowment

Although nephrogenesis may continue for several weeks after preterm birth, postnatal nephron formation appears structurally abnormal and quantitatively insufficient to compensate for gestational interruption. Histological studies demonstrate malformed glomeruli, reduced cortical nephron density, and impaired vascular organization, indicating that extrauterine nephrogenesis does not fully replicate intrauterine development [11]. Even when nephron formation persists beyond 40 postnatal days, structural dysmaturation remains evident [12].

Accordingly, individuals born preterm consistently exhibit lower nephron endowment compared with those born at term. Reduced nephron number limits renal functional reserve and increases susceptibility to secondary stressors such as hypertension, obesity and AKI [3]. Experimental models show that kidneys with low nephron density develop accelerated glomerulosclerosis when exposed to hemodynamic stress, supporting a causal link between nephron endowment and long-term vulnerability [3].

Population-based studies corroborate these mechanistic findings. Large national cohorts demonstrate that preterm birth is independently associated with increased CKD risk from childhood into mid-adulthood [1], with risk rising as gestational age decreases, consistent with a dose–response relationship [5]. These epidemiologic data reinforce the concept that kidney disease risk in prematurity originates from developmental structural constraints.

Reduced nephron endowment also influences renal hemodynamics, sodium handling and intrarenal oxygenation, further predisposing to hypertension and progressive kidney injury [8]. Together, these observations align with the developmental origins of health and disease framework, whereby early disturbances in kidney development exert lifelong effects on renal structure and function [9].

### 2.3. Compensatory Mechanisms: Glomerular Hypertrophy and Hyperfiltration

To maintain adequate global filtration capacity in the setting of reduced nephron number, remaining nephrons undergo compensatory hypertrophy and increased single-nephron GFR, a process known as hyperfiltration [4]. While initially adaptive, sustained hyperfiltration exposes glomeruli to increased mechanical and metabolic stress, promoting endothelial dysfunction and podocyte injury [3].

Over time, these maladaptive responses result in mesangial expansion, basement membrane thickening, and progressive glomerulosclerosis, ultimately accelerating nephron loss [2]. As nephron number continues to decline, the filtration burden on remaining glomeruli further increases, establishing a self-perpetuating cycle of injury. This sequence of events is believed to account for the heightened prevalence of hypertension, proteinuria, and reduced renal reserve observed in individuals born preterm [5].

Clinical studies in children and adolescents born extremely preterm demonstrate reduced kidney volumes, elevated albumin excretion and abnormal ambulatory blood pressure profiles, even in the absence of overt kidney disease [13,14]. These findings suggest that structural and functional renal alterations may be present long before clinically apparent CKD develops, highlighting the importance of early detection strategies.

Importantly, hyperfiltration-associated injury may be exacerbated by postnatal growth acceleration and metabolic stress, particularly during periods of rapid somatic growth such as infancy and puberty [8]. These physiological transitions may further strain already vulnerable nephrons, accelerating progression toward clinically significant kidney dysfunction. This compensatory response serves as a central adaptive pathway linking reduced nephron endowment to long-term renal vulnerability.

### 2.4. The Multiple-Hit Hypothesis in Preterm Kidney Injury

While reduced nephron endowment constitutes the foundational “first hit,” maternal metabolic and nutritional factors represent upstream modifiers of renal vulnerability that precede preterm birth itself. Conditions such as maternal diabetes, obesity and suboptimal maternal nutrition are independently associated with altered fetal nephron development, impaired placental perfusion, and epigenetic programming of renal and vascular pathways [15]. These factors may simultaneously increase the risk of preterm delivery and predispose the neonate to reduced nephron endowment and early renal dysfunction, thereby amplifying the impact of subsequent postnatal insults [16]. In this context, developmental nephron deficit is not an isolated structural finding but a determinant of reduced renal reserve that increases susceptibility to later hemodynamic and inflammatory stress.

In contrast, caffeine citrate has emerged as a potentially nephroprotective intervention in preterm infants. Beyond its established role in reducing apnea of prematurity and the need for mechanical ventilation, caffeine may attenuate systemic inflammation, improve renal hemodynamics and reduce the incidence of neonatal AKI [17]. Evidence from the BABYCCINO trial and subsequent analyses suggests that early caffeine exposure is associated with improved renal outcomes and lower risk of AKI, likely mediated through reduced ventilator exposure and stabilization of the cardiorespiratory system [18]. These observations support the concept that modulation of early postnatal exposures may influence long-term renal trajectories in a kidney already constrained by incomplete nephrogenesis.

Perinatal inflammatory stress may further impair renal maturation by disrupting nephron differentiation and microvascular development [10]. Moreover, neonatal conditions such as bronchopulmonary dysplasia and growth restriction often coexist with renal vulnerability, reflecting shared pathways of developmental dysregulation [6]. These interactions illustrate that kidney injury in individuals born preterm rarely arises from a single mechanism but rather from cumulative insults superimposed on developmental limitations. Importantly, impaired microvascular development and inflammatory activation during this period may contribute to later endothelial dysfunction, altered sodium handling, and elevated blood pressure observed in adolescence and adulthood.

Longitudinal studies indicate that growth restriction, maternal preeclampsia and postnatal metabolic disturbances further amplify long-term renal risk among very low birth weight (VLBW) infants [19]. Thus, kidney disease susceptibility reflects a complex interplay between prenatal programming, neonatal exposures, and later-life metabolic and hemodynamic stressors. This interaction helps explain why measurable outcomes such as increased albuminuria, higher ambulatory blood pressure, reduced kidney volume, and subtle decline in cystatin C–based eGFR may emerge years after the initial developmental insult.

This multiple-hit model highlights the clinical importance of minimizing modifiable renal insults throughout early life. Strategies such as careful monitoring of kidney function, avoidance of nephrotoxic drugs, prevention of AKI, and early management of hypertension may help preserve renal reserve in this high-risk population [7]. Importantly, this framework also highlights opportunities for early intervention during childhood, when disease trajectories may still be modifiable.

**Figure 1:**
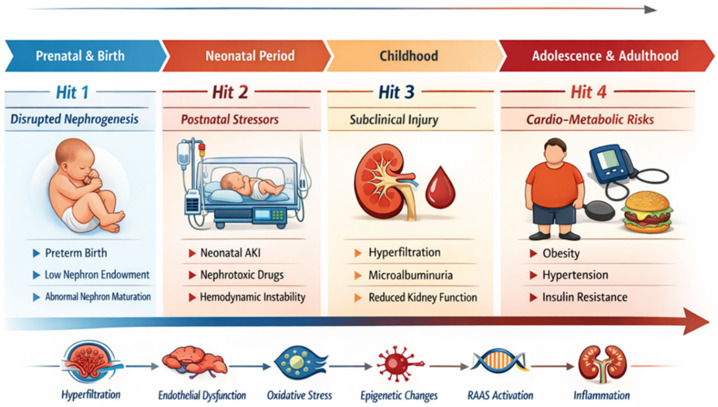
Life-course multiple-hit model of renal vulnerability after preterm birth. Interrupted nephrogenesis leads to reduced nephron endowment (first hit), followed by neonatal insults such as acute kidney injury and nephrotoxic exposures (second hit). Persistent hyperfiltration and subclinical renal injury during childhood constitute additional stressors (third hit), while cardiometabolic risk factors in adolescence and adulthood further accelerate progression toward chronic kidney disease (fourth hit).

Overall, these data support a life-course, multiple-hit model of renal vulnerability after preterm birth. Reduced nephron endowment constitutes the initial developmental insult, followed by superimposed neonatal exposures such as AKI, nephrotoxic medications, and mechanical ventilation, and subsequently amplified by persistent hyperfiltration, hypertension, and cardiometabolic stress across childhood and adulthood. These interacting insults progressively reduce renal reserve and increase long-term CKD susceptibility (Figure 1). In this stepwise framework, each “hit” contributes to a transition from structural nephron deficit to functional impairment, ultimately translating developmental vulnerability into clinically detectable chronic kidney disease risk.

Although the multiple-hit paradigm may also apply to term-born infants exposed to hemodynamic instability or nephrotoxic therapies, preterm birth is distinguished by the presence of an initial developmental nephron deficit resulting from interrupted nephrogenesis. This reduced nephron endowment constitutes a unique biological substrate that amplifies the impact of subsequent postnatal insults and differentiates prematurity from renal stress occurring in structurally mature kidneys.

## 3. Pathophysiological Mechanisms Linking Preterm Birth to Kidney Injury

### 3.1. Hemodynamic and Structural Changes

Preterm birth reduces nephron endowment and therefore increases the filtration workload per remaining nephron, initiating adaptive hyperfiltration that may become maladaptive over time [4]. This compensatory rise in single-nephron GFR leads to sustained intraglomerular hypertension and mechanical strain on the filtration barrier [2]. The hyperfiltration paradigm provides a coherent mechanistic link between developmental oligonephropathy and later CKD risk across the life course [20].

Antenatal corticosteroids do not directly promote renal maturation or nephrogenesis. Their beneficial effects on kidney outcomes are instead mediated indirectly through enhanced pulmonary maturation and surfactant production, resulting in reduced severity of respiratory distress syndrome. Improved respiratory adaptation leads to less exposure to invasive mechanical ventilation, lower hemodynamic instability, and reduced ventilator-induced lung injury, collectively attenuating systemic and renal inflammation. By limiting hypoxia, circulatory fluctuations, and inflammatory burden during the critical early neonatal period, antenatal steroids may indirectly mitigate secondary renal insults in preterm infants [10,17].

In the neonatal intensive care setting, patent ductus arteriosus (PDA) and its pharmacologic management with non-steroidal anti-inflammatory drugs (NSAIDs) constitute a frequent and clinically relevant renal stressor. NSAID exposure, combined with altered renal perfusion and ductal shunting, may further compromise glomerular filtration and tubular integrity in preterm infants, thereby amplifying hemodynamic strain on an already nephron-deficient kidney [21]. Mechanical ventilation contributes to renal injury through systemic inflammation, neurohormonal activation, and impaired renal perfusion, linking ventilator-induced lung injury to secondary kidney damage within the lung–kidney axis.

Persistently elevated glomerular pressure triggers structural remodeling, including glomerular hypertrophy, mesangial expansion, and thickening of the glomerular basement membrane, which progressively compromises perm-selectivity [3]. Podocyte stress and endothelial dysfunction further destabilize the filtration barrier, promoting albumin leakage and progressive glomerulosclerosis [2]. Importantly, both human and experimental observations suggest that nephron formation may continue briefly after preterm birth but often under atypical conditions that favor dysmaturation rather than full compensation [22].

Renal injury following prematurity also involves the renal microvasculature. Perinatal inflammatory stress and disrupted angiogenic signaling may impair microvascular development, leading to reduced peritubular capillary density and predisposing the kidney to chronic hypoxia [10]. Chronic hypoxic stress activates profibrotic pathways, including transforming growth factor-β (TGF-β) signaling, and accelerates interstitial fibrosis and tubular atrophy [9]. These interstitial and vascular mechanisms synergize with glomerular hyperfiltration to drive progressive nephron loss.

Subclinical structural alterations may be detectable before overt decline in eGFR. Adolescents born extremely preterm have been shown to exhibit reduced kidney volumes alongside higher prevalence of albuminuria and elevated blood pressure, supporting early structural–functional coupling [13]. Similar cohorts demonstrate associations between kidney volume, kidney function indices, and ambulatory blood pressure patterns, suggesting early hemodynamic imprinting [14]. Longitudinal pediatric follow-up of extremely low birth weight (ELBW) and VLBW infants further suggests that reduced renal size and early functional abnormalities may persist into school age, consistent with early-onset vulnerability [23].

At the population level, meta-analytic evidence supports that preterm birth is associated with lower GFR, higher blood pressure, and increased albuminuria, consistent with a hyperfiltration–injury pathway [24]. These data align with large national cohorts demonstrating increased CKD risk from childhood into mid-adulthood, suggesting that structural developmental constraints translate into clinically meaningful renal outcomes [1]. Collectively, structural remodeling and hemodynamic dysregulation appear to represent central pathways through which developmental nephron deficits evolve into progressive kidney injury.

### 3.2. Metabolic and Oxidative Stress

Oxidative stress is increasingly recognized as a key amplifier of renal vulnerability in individuals born preterm. Premature kidneys have immature antioxidant defenses, rendering renal parenchyma more susceptible to reactive oxygen species generated during hypoxia, ischemia, infection and systemic inflammation [11]. In the context of prematurity, this vulnerability is particularly relevant because these exposures frequently occur during an active phase of ongoing nephron maturation. Experimental models demonstrate that prematurity-related disturbances combined with metabolic stress (e.g., hyperglycemia) increase oxidative burden and disrupt nephron maturation, supporting a mechanistic link between neonatal exposures and later renal susceptibility [11]. Such disruption may impair glomerular differentiation, reduce microvascular integrity and predispose to long-term reductions in functional nephron mass, thereby contributing to a structurally constrained renal reserve rather than solely promoting hyperfiltration. In preterm infants, these processes occur during an active phase of nephrogenesis, rendering the kidney uniquely susceptible compared with structurally mature term-born kidneys.

Exposures during neonatal intensive care may further intensify oxidative injury during a critical developmental window. Hyperoxia has been shown to impair nephrogenesis and promote long-term structural vulnerability, suggesting that oxygen-related stress may function as an early “second hit” in susceptible kidneys [25]. Importantly, these experimental findings are consistent with clinical observations of smaller kidney volumes, altered renal perfusion indices and early markers of endothelial dysfunction in cohorts born extremely preterm. Such translational concordance strengthens the proposed link between neonatal oxidative stress and later structural and vascular alterations. These vascular and microcirculatory changes likely interact with adaptive hemodynamic responses, further increasing susceptibility to subsequent renal injury. This framework aligns with clinical observations that preterm infants frequently experience hemodynamic instability and inflammatory states that can magnify oxidative stress and renal injury [6].

Metabolic load later in childhood and adolescence can compound the initial nephron deficit. Rapid catch-up growth together with early adiposity increases metabolic demand and glomerular workload, adding to cumulative renal stress across multiple pathways [8]. In individuals born preterm, this additional load acts upon a developmentally reduced nephron pool, thereby amplifying vulnerability compared with peers born at term. Fetal and early postnatal growth restriction further compound the renal consequences of prematurity. Intrauterine growth restriction is associated with reduced nephron number, smaller kidney volumes and altered tubular development, while inadequate postnatal growth may limit compensatory renal maturation. Together, these factors exacerbate hyperfiltration stress, thereby increasing susceptibility to hypertension and CKD later in life [8,19]. This interaction between early oxidative injury and later metabolic stress provides a biologically plausible explanation for the emergence of albuminuria and elevated blood pressure during adolescence in this population.

Renal energy metabolism and mitochondrial resilience may also be altered by early-life stress. Developmental programming mechanisms can impair cellular repair capacity following injury, shifting healing toward fibrosis rather than regeneration [9]. Mitochondrial dysfunction in tubular epithelial cells may reduce adenosine triphosphate (ATP) availability, impair sodium handling and promote profibrotic signaling pathways, thereby linking early oxidative injury with later interstitial fibrosis. In the setting of prematurity, where antioxidant defenses and vascular maturation are incomplete, mitochondrial susceptibility may be further accentuated. These mechanisms suggest that tubular metabolic vulnerability and microvascular dysfunction operate alongside glomerular hyperfiltration within a broader injury network. Consistent with this vulnerability, neonatal AKI represents a potent stressor in preterm infants and is associated with downstream risk of adverse kidney outcomes, highlighting AKI as a clinically important mediator and modifiable target [15]. Thus, oxidative and metabolic stress pathways connect neonatal intensive care unit (NICU)-era insults and later-life exposures into a cumulative injury trajectory, ultimately translating early redox imbalance into measurable long-term functional impairment. Importantly, these pathways are interpreted here within the specific developmental context of interrupted nephrogenesis rather than as generic mechanisms of kidney injury.

### 3.3. Epigenetic and Developmental Programming Effects

Beyond hemodynamics and oxidative injury, developmental programming and epigenetic mechanisms are likely to contribute to persistent renal vulnerability after preterm birth. The DOHaD framework proposes that early-life disturbances can induce long-lasting changes in gene expression related to nephron development, vascular tone and inflammation, shaping disease susceptibility across the lifespan [9]. In prematurity, such programming may reflect both interrupted nephrogenesis and the abrupt transition to extrauterine exposures during a sensitive developmental period [2].

Experimental and translational evidence indicates that developmental stress can alter pathways involved in renin–angiotensin signaling, oxidative stress responses and extracellular matrix regulation, thereby priming kidneys toward hypertension, albuminuria and fibrosis when later exposures occur [8]. Perinatal inflammatory stress may be particularly relevant, as it can “prime” immune and profibrotic pathways and increase susceptibility to CKD beyond oligonephropathy alone [10]. This inflammatory programming plausibly contributes to exaggerated fibrotic responses after modest secondary insults, independently of hyperfiltration-driven mechanisms. Such effects may be particularly relevant in preterm populations, in whom early immune and vascular maturation is developmentally incomplete.

Although not systematically evaluated in large epidemiologic cohorts, connective tissue abnormalities and extracellular matrix dysregulation may theoretically further influence postnatal nephrogenesis and renal vascular maturation. Altered matrix composition or structural support during ongoing kidney development could modify nephron differentiation, microvascular stability, and long-term fibrotic response. However, direct clinical evidence linking connective tissue dysplasia specifically to prematurity-associated CKD trajectories remains limited.

Programming effects may also extend to vascular biology and systemic blood pressure regulation. Altered endothelial function and increased vascular stiffness may contribute to elevated blood pressure, which in turn augments intraglomerular stress and accelerates functional decline [8]. These findings support the concept that renal vulnerability after prematurity reflects coordinated cardio-renal remodeling rather than isolated glomerular adaptation. Cohort studies in adolescents born extremely preterm demonstrate clustering of albuminuria, elevated ambulatory blood pressure and reduced kidney volumes, consistent with a programmed cardio-renal phenotype [13]. Similar pediatric cohorts assessed with ambulatory monitoring report patterns compatible with early vascular programming and sustained renal hemodynamic stress [14].

Importantly, developmental programming mechanisms also imply potential windows for prevention. Contemporary expert perspectives emphasize nephron preservation and early-life risk mitigation as priorities, including minimization of nephrotoxins, prevention and prompt recognition of AKI, as well as systematic surveillance of blood pressure and albuminuria [7]. Clinical reviews targeted at neonatal–pediatric practice similarly highlight the need for structured follow-up pathways that connect neonatal exposures to long-term kidney monitoring [26]. Together, these insights support the view that prematurity-related kidney risk is not inevitable but may be modifiable through timely identification and targeted preventive care.

## 4. Long-Term Renal Outcomes: Clinical Evidence Across the Life Course

### 4.1. Gestational Age–Stratified Renal Risk

Renal consequences following preterm birth are not uniform but demonstrate a graded relationship across gestational age categories. Individuals born extremely preterm (<28 weeks) consistently exhibit the highest risk of reduced nephron endowment, smaller kidney volumes, elevated blood pressure and later CKD development. Those born very preterm (28–32 weeks) show intermediate risk, whereas moderate-to-late preterm infants (32–36 weeks) generally demonstrate subtler alterations, although epidemiologic data suggest that vulnerability persists along a continuum of gestational maturity. Large population-based registries and meta-analyses support this dose–response relationship, indicating that decreasing gestational age correlates with increasing long-term renal risk [1,5]. This stratification underscores that nephron deficit severity likely parallels gestational age at birth, thereby influencing the magnitude of subsequent hyperfiltration stress and CKD susceptibility.

### 4.2. CKD Development

The clinical manifestations described below represent the cumulative expression of the developmental and postnatal mechanisms previously described, translating structural nephron deficit and molecular vulnerability into measurable long-term renal outcomes. Robust epidemiologic evidence now links preterm birth with an increased lifetime risk of CKD. In a landmark Swedish registry including more than four million individuals, those born preterm exhibited nearly double the risk of CKD by mid-adulthood, with a graded relationship across gestational age categories and the highest risk observed among those born extremely preterm [1]. This graded association supports the concept that the magnitude of nephron endowment reduction correlates with long-term renal risk. Importantly, even early-term birth was associated with modestly elevated risk, suggesting that renal vulnerability exists along a continuum of gestational maturity rather than being restricted to VLBW infants.

Subsequent systematic reviews and meta-analyses have confirmed these findings, consistently demonstrating associations between prematurity and lower eGFR as well as higher prevalence of CKD in childhood and young adulthood, particularly when gestational age is below 32 weeks [5]. More recent pooled analyses report average reductions in eGFR of approximately 10–15 mL/min/1.73 m^2^ among adolescents and young adults born preterm, accompanied by higher cystatin C levels, indicating early impairment of renal filtration capacity [27]. These functional alterations are consistent with progressive nephron loss and sustained hyperfiltration stress described in the preceding mechanistic sections. Importantly, these interpretations are supported by longitudinal registry data and pooled analyses rather than inferred solely from experimental models [1,5,27].

Beyond population-level CKD risk, prematurity may also modify the clinical course of intrinsic kidney diseases. In a large cohort spanning both pediatric and adult patients with glomerular disorders, preterm birth was independently associated with more rapid progression and worse renal outcomes, suggesting that reduced nephron reserve may amplify vulnerability once a primary renal insult occurs [28]. Collectively, these data support the interpretation that prematurity constitutes a substrate of limited renal reserve rather than merely a statistical association. Accordingly, prematurity may function not only as a background risk factor for CKD but also as a disease modifier influencing long-term prognosis following secondary renal injury.

Conceptually, these observations align with the developmental origins of health and disease framework, in which early-life perturbations shape lifelong susceptibility to chronic conditions. From this perspective, renal programming after preterm birth reflects the cumulative effects of impaired nephrogenesis, postnatal exposures and adaptive hemodynamic stress that together establish a trajectory toward progressive nephron loss [29,30]. Importantly, the detection of reduced renal clearance during adolescence indicates that this trajectory is not theoretical but clinically measurable years before overt CKD becomes apparent. Clinical data showing reduced renal clearance already detectable in adolescence reinforce that this trajectory may be clinically measurable long before overt CKD becomes apparent [31]. Importantly, the renal trajectory of individuals born preterm may be further modified by the presence of congenital anomalies of the kidney and urinary tract (CAKUT) or genetically determined kidney diseases. In such contexts, prematurity does not necessarily constitute the primary etiologic factor but may exacerbate pre-existing structural or molecular vulnerability by further limiting nephron reserve. The coexistence of congenital abnormalities and developmental nephron deficit may accelerate progression toward CKD, while inherited disorders affecting podocyte or tubular function may manifest earlier or more aggressively in the setting of reduced renal reserve. Thus, prematurity should be interpreted not only as an independent risk factor but also as a disease modifier in children with underlying renal pathology.

### 4.3. Hypertension and Altered Sodium Handling

Elevated blood pressure represents one of the most consistent and earliest clinical sequelae observed among individuals born preterm. Multiple longitudinal cohorts demonstrate higher systolic and diastolic blood pressure values in children, adolescents and young adults born prematurely compared with term-born controls, typically in the range of 2–4 mmHg [13,14]. Although modest at the individual level, these shifts translate into substantial population-level increases in lifetime cardiovascular and renal risk.

Mechanistically, reduced nephron number increases single-nephron sodium reabsorption and promotes chronic activation of the Renin–Angiotensin–Aldosterone system (RAAS), leading to persistent elevations in systemic vascular resistance. Sympathetic nervous system activation and impaired baroreceptor sensitivity may further contribute to sustained hypertension. Experimental studies demonstrating attenuation of glomerular injury with RAAS blockade in models of reduced nephron endowment support a causal link between developmental nephron deficit and later hypertension [8].

Long-term cohort data extend these observations into adulthood. In the Pelotas 1993 birth cohort, individuals born preterm exhibited altered growth trajectories and higher adult blood pressure, reinforcing that cardiovascular consequences of prematurity persist decades beyond the neonatal period [32]. Clinical syntheses further emphasize that hypertension frequently precedes overt decline in eGFR and likely contributes substantially to progressive renal injury over time [33].

### 4.4. Albuminuria and Early Glomerular Injury

Microalbuminuria is increasingly recognized as an early marker of glomerular stress in populations born preterm. Studies in school-aged children and adolescents demonstrate urinary albumin-to-creatinine ratios that are significantly higher than in term-born peers, even when eGFR remains within normal limits [13]. Persistent low-grade albuminuria reflects endothelial dysfunction and increased glomerular permeability, signaling early disruption of the filtration barrier.

Cystatin C provides additional sensitivity for detecting subtle impairments in renal filtration that may not be captured by creatinine-based estimates, particularly in children with lower muscle mass. Elevated cystatin C concentrations have been documented in preterm cohorts as early as preschool age, preceding measurable declines in creatinine-derived eGFR [31]. These findings support the notion that functional impairment may begin early and remain clinically silent for prolonged periods.

At the cellular level, recent mechanistic synthesis implicate podocyte vulnerability as a central pathway linking prematurity to long-term CKD. Structural and metabolic stress imposed on podocytes during postnatal adaptation may predispose to progressive loss of filtration barrier integrity, promoting albuminuria and accelerating glomerulosclerosis over time [34]. This cellular susceptibility provides a biologically plausible explanation for why prematurity may amplify risk of glomerular disease progression later in life.

### 4.5. Reduced Kidney Volume and Lifelong Functional Deficits

Structural imaging consistently reveals smaller renal volumes in individuals born preterm, reflecting reduced nephron endowment. Ultrasound and magnetic resonance imaging (MRI) studies in childhood and adolescence demonstrate decreased total kidney volume, thinner cortices, and altered cortical-to-medullary ratios compared with term-born controls [14,23]. These differences often persist despite apparently normal serum creatinine, indicating that structural deficits may precede functional decline.

Diffusion-weighted MRI further demonstrates reduced cortical perfusion and restricted diffusion patterns consistent with decreased nephron density and microvascular alterations. When paired with functional measures showing reduced clearance and elevated cystatin C, these imaging findings support a phenotype of compensated nephron loss that may remain clinically silent until additional stressors supervene [31]. Such stressors include obesity, hypertension, pregnancy and nephrotoxic exposures, which may precipitate more rapid functional decline in kidneys with limited reserve.

### 4.6. Cardiometabolic Interactions

Renal outcomes after preterm birth are closely intertwined with cardiovascular and metabolic sequelae. Numerous cohorts demonstrate higher rates of insulin resistance, dyslipidemia and arterial stiffness among individuals born preterm, beginning in childhood and persisting into adulthood [32,33]. These abnormalities contribute to increased glomerular perfusion pressure, endothelial dysfunction and progressive microvascular injury within the kidney.

Insulin resistance promotes hyperfiltration, while dyslipidemia accelerates atherosclerotic changes within renal microvasculature, further compounding nephron loss. Together, these mechanisms establish a cardio-renal-metabolic phenotype rooted in early developmental programming [29]. Recognition of this interconnected pathophysiology underscores the need for integrated surveillance strategies that address both renal and cardiovascular risk in preterm survivors.

### 4.7. Modifying Factors: Neonatal AKI, Sex and Genetic Susceptibility

Neonatal AKI represents a major modifier of long-term renal outcomes following prematurity. Preterm infants experience high rates of AKI during neonatal intensive care, often in the context of hemodynamic instability, sepsis, or nephrotoxic medication exposure. Evidence suggests that AKI superimposed on reduced nephron endowment may substantially accelerate progression toward CKD [35]. Follow-up studies of adolescents born extremely preterm indicate that renal outcomes may be worse among those with a history of neonatal AKI, even when early recovery appeared complete [36].

Sex differences are also emerging, with male neonates exhibiting higher rates of neonatal AKI and potentially greater susceptibility to hypoxic and inflammatory injury, whereas females may display higher prevalence of hypertension during adolescence. Hormonal modulation of vascular tone and renin–angiotensin signaling may contribute to these divergent trajectories, although definitive mechanisms remain under investigation [30].

Genetic susceptibility likely contributes further to interindividual variability. Variants affecting nephron development, podocyte integrity and blood pressure regulation may modulate long-term renal resilience after prematurity. While large genome-wide studies in preterm cohorts remain limited, emerging evidence suggests polygenic contributions to differences in GFR and blood pressure trajectories across childhood and adulthood [29].

### 4.8. Clinical and Public Health Implications

The burden of prematurity-related renal dysfunction carries important clinical and public health implications. CKD often remains asymptomatic until advanced stages, making early identification within this high-risk population essential. Routine monitoring of blood pressure, albuminuria and renal function using both creatinine and cystatin C should be incorporated into long-term follow-up of individuals born preterm, particularly those with a history of neonatal AKI [35,36].

From a population perspective, even small average reductions in nephron reserve across millions of preterm survivors translate into substantial long-term CKD burden. Preventive strategies addressing maternal health, neonatal care and equitable access to long-term surveillance may therefore yield significant societal benefits [1,29]. Framing kidney health after prematurity within a life-course approach provides opportunities for early intervention aimed at preserving renal reserve and mitigating progression toward CKD.

## 5. Current Clinical Management and Preventive Strategies

### 5.1. Antenatal Interventions to Support Nephrogenesis

Antenatal care plays a pivotal role in shaping renal health trajectories among infants at risk of preterm birth. Administration of antenatal corticosteroids accelerates fetal organ maturation and improves neonatal survival, indirectly protecting renal adaptation by reducing respiratory and cardiovascular instability after birth [33]. Although corticosteroids do not increase nephron number, improved systemic stability may limit secondary hypoxic and hemodynamic insults to the immature kidney.

Maternal conditions that impair placental perfusion or promote systemic inflammation, including preeclampsia, chronic hypertension, diabetes, malnutrition and intrauterine infection, are strongly associated with impaired fetal nephrogenesis and increased susceptibility to neonatal AKI [29,37]. Early identification and aggressive management of these conditions may therefore reduce the magnitude of nephron deficit and attenuate subsequent renal vulnerability. Inflammatory and oxidative intrauterine exposures may also prime renal tissue for exaggerated postnatal responses to ischemia and nephrotoxins, reinforcing the importance of minimizing prenatal stressors whenever possible [10].

From a public health perspective, prevention of preterm delivery remains the most effective strategy to preserve nephron endowment. Population-based data demonstrate that reductions in prematurity rates are likely to translate into meaningful decreases in long-term CKD burden, highlighting the importance of maternal health policies and access to high-quality prenatal care [1].

### 5.2. Neonatal Intensive Care Approaches

After preterm birth, neonatal intensive care practices exert substantial influence on long-term renal outcomes. Maintenance of hemodynamic stability through individualized fluid management and avoidance of prolonged hypotension reduces ischemic stress on immature nephrons and supports adaptive postnatal renal maturation [21]. Both fluid overload and inadequate perfusion may exacerbate renal injury, emphasizing the need for hemodynamic targets tailored to gestational age and clinical condition.

Neonatal AKI is common among preterm infants and represents a major determinant of long-term kidney health. A recent global meta-analysis indicates that a substantial proportion of preterm neonates develop AKI during NICU hospitalization, underscoring the scale of this clinical problem [38]. AKI superimposed on reduced nephron endowment may accelerate progression toward CKD, even when apparent short-term recovery occurs [35].

Avoidance of nephrotoxic exposures is a cornerstone of kidney-protective neonatal care. Aminoglycoside antibiotics, nonsteroidal anti-inflammatory drugs, antifungal agents and iodinated contrast media are frequently used in critically ill neonates and may substantially increase AKI risk when administered cumulatively [39]. Implementation of nephrotoxin stewardship programs, medication alerts and multidisciplinary monitoring protocols has been associated with reduced AKI incidence and lower cumulative nephrotoxic burden in NICU settings [21].

In cases of severe or persistent renal failure, renal replacement therapies adapted for VLBW infants may be required. Peritoneal dialysis remains the most widely used modality in this population because of its technical feasibility and favorable hemodynamic profile, enabling metabolic stabilization while minimizing additional renal stress [15].

### 5.3. Avoidance of Nephrotoxic Agents Across Childhood

Renal vulnerability persists beyond NICU discharge. Children born preterm often require repeated hospitalizations and prolonged pharmacologic treatments, increasing cumulative exposure to potentially nephrotoxic medications throughout early childhood [35]. Restricting nephrotoxic agents to situations where safer alternatives are unavailable remains an essential preventive strategy.

Clinical protocols incorporating scheduled renal function monitoring, urine output assessment and electronic alerts for high-risk drug combinations facilitate early detection of medication-related renal injury. Prompt dose adjustment according to renal function and early discontinuation of nephrotoxic agents can reduce the likelihood of irreversible nephron loss [39]. Education of caregivers and primary care providers regarding avoidance of over-the-counter nephrotoxins, particularly nonsteroidal anti-inflammatory drugs, is equally important as children transition into outpatient care.

Given the limited nephron reserve characteristic of individuals born preterm, even transient episodes of drug-induced renal injury may have disproportionate long-term consequences compared with term-born peers, supporting the need for heightened vigilance throughout childhood and adolescence [36].

### 5.4. Long-Term Monitoring, Biomarkers and Risk Stratification

Structured long-term surveillance is essential for early detection of evolving kidney dysfunction in individuals born preterm. Traditional assessment relies on serum creatinine (sCr), which is often confounded by maternal levels and the infant’s low muscle mass. Consequently, cystatin C has emerged as a superior marker because it is produced at a constant rate by nucleated cells and is largely independent of body composition or placental transfer, providing a more reliable estimation of GFR from birth [40,41].

Beyond GFR, biomarkers such as neutrophil gelatinase-associated lipocalin (NGAL) and kidney injury molecule-1 (KIM-1) offer critical diagnostic value by distinguishing functional hemodynamic shifts from true structural tubular injury [40]. Unlike sCr, which rises only after significant functional loss, these injury markers reflect immediate tubular stress and ongoing subclinical injury, enabling early detection of acute tubular necrosis, often days before sCr elevations [42]. The biological sources, clinical significance and potential advantages of circulating and urinary biomarkers in the neonatal and pediatric setting are summarized in Table 1, highlighting their role in risk stratification beyond creatinine alone.

Imaging modalities also provide valuable structural information; for instance, the kidney volume-to-birth-weight ratio has been proposed as a practical surrogate marker of nephron endowment in ELBW infants [43]. Consensus recommendations emphasize the need for structured follow-up after neonatal AKI, including periodic assessment of blood pressure together with albuminuria during adolescence and adulthood [33,44]. Ultimately, risk stratification should integrate gestational age, birth weight, AKI episodes and cumulative nephrotoxic exposure to enable personalized surveillance and early intervention [36].

### 5.5. Lifestyle, Nutrition and Pharmacologic Management in Adolescence and Adulthood

Lifestyle interventions represent an important component of renal preservation in populations born preterm. Balanced nutrition, maintenance of healthy body weight and moderation of dietary sodium intake reduce cardiovascular and intraglomerular pressure stress, thereby slowing progressive nephron loss [29]. Regular physical activity improves insulin sensitivity and blood pressure control, mitigating clustering of cardio-renal risk factors.

Experimental approaches aimed at prolonging postnatal nephrogenesis through optimized nutrition, growth factor modulation and careful control of oxygen exposure have been proposed, although translation into routine clinical practice remains limited [25]. Nonetheless, optimized early nutrition supports somatic growth and may indirectly benefit renal maturation by improving overall metabolic stability.

Pharmacologic therapy becomes necessary when hypertension, albuminuria, or declining GFR is detected. Agents targeting the RAAS, including angiotensin-converting enzyme inhibitors and angiotensin receptor blockers, reduce intraglomerular hypertension and proteinuria and remain central to renoprotective strategies [33]. Early initiation of therapy in appropriately selected individuals may delay progression toward overt CKD.

Special consideration is warranted during pregnancy in women born preterm, as reduced renal reserve may predispose to hypertensive disorders of pregnancy and accelerate post-partum renal decline. Multidisciplinary obstetric and nephrologic care is therefore recommended during reproductive years to minimize maternal and fetal complications [30].

**Figure 2:**
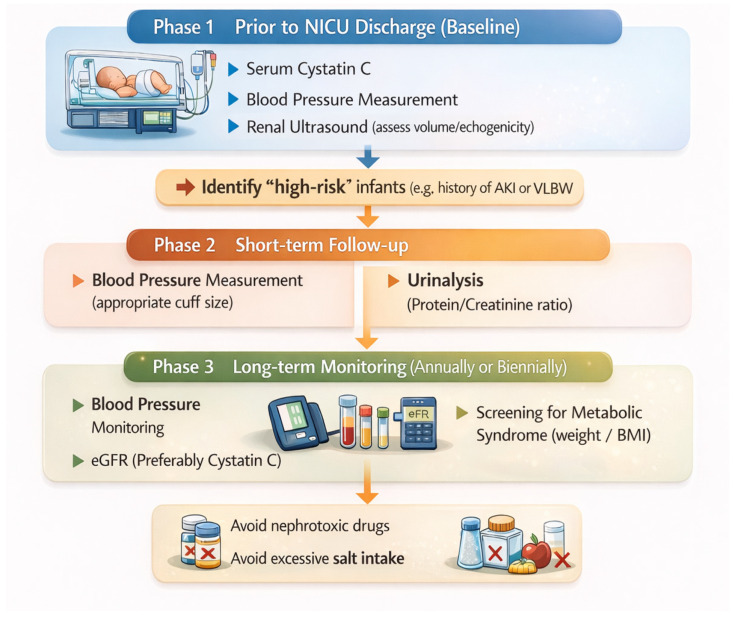
Structured follow-up algorithm for kidney monitoring after preterm birth. Monitoring is structured into three phases: baseline assessment prior to NICU discharge, early post-discharge follow-up and long-term surveillance. The algorithm aims to identify high-risk infants and enable early detection of hypertension, albuminuria, and declining renal function to support timely preventive interventions.

Taken together, these preventive and monitoring strategies support the need for a structured, phase-based follow-up approach that integrates neonatal risk factors with ongoing assessment of blood pressure, albuminuria and renal function across childhood and adulthood. Such an approach enables early identification of high-risk individuals and timely implementation of renoprotective interventions before irreversible loss of renal reserve occurs. A practical framework for longitudinal kidney health surveillance after preterm birth, spanning NICU discharge, early post-discharge follow-up and long-term monitoring, is summarized in Figure 2.

## 6. Emerging Therapeutic Targets and Research Directions

### 6.1. Hemodynamic and Cellular Stress Pathways

#### 6.1.1. RAAS Dysregulation and Glomerular Hypertension

Reduced nephron endowment following preterm birth leads to adaptive activation of the RAAS to maintain systemic and intraglomerular hemodynamics [2,20]. Persistent RAAS upregulation promotes efferent arteriolar vasoconstriction, elevated intraglomerular pressure, and sustained hyperfiltration, thereby accelerating podocyte stress, mesangial expansion and glomerulosclerosis [3]. Experimental models further suggest that early-life RAAS activation induces long-lasting epigenetic and transcriptional changes in renal vascular and tubular compartments, reinforcing susceptibility to later hypertensive and proteinuric injury [9,45].

#### 6.1.2. TGF-β Signaling, Fibrogenesis and Maladaptive Repair

Postnatal kidney injury in preterm infants, particularly in the context of AKI and inflammatory exposures, is associated with activation of TGF-β signaling pathways that promote fibrogenic responses [10]. TGF-β induces fibroblast activation, epithelial–mesenchymal transition, and extracellular matrix accumulation, leading to tubular atrophy and interstitial fibrosis [20]. Recurrent or subclinical injury during periods of ongoing renal maturation may therefore bias repair toward maladaptive fibrotic remodeling rather than regenerative recovery, accelerating CKD progression later in life [3,6].

#### 6.1.3. Impaired Vascular Endothelial Growth Factor (VEGF)-Mediated Angiogenesis and Endothelial Dysfunction

Normal nephrogenesis requires tightly coordinated glomerular and peritubular capillary development mediated by VEGF signaling [45]. Prematurity disrupts this angiogenic program, resulting in reduced microvascular density and long-term endothelial dysfunction [11,22]. Subsequent exposures such as hypoxia, oxidative stress, and nephrotoxic medications may further compromise microvascular integrity, promoting chronic renal hypoxia and progressive nephron dropout [10,25].

#### 6.1.4. Mitochondrial Dysfunction and Metabolic Stress Pathways

Emerging evidence indicates that mitochondrial biogenesis and oxidative phosphorylation capacity are incompletely matured in the preterm kidney [45]. Postnatal oxidative stress, hypoxic episodes, and inflammatory signaling can induce persistent mitochondrial injury and metabolic reprogramming in tubular epithelial cells, leading to impaired ATP production and heightened vulnerability to secondary insults [11,33]. These mechanisms may link early developmental disruption with later susceptibility to cardiometabolic comorbidities and accelerated CKD progression [3,29].

### 6.2. Antioxidant Strategies and Redox Modulation

Oxidative stress plays a central role in mediating renal injury associated with prematurity. Preterm infants exhibit immature antioxidant systems and are frequently exposed to hyperoxia, inflammation, and ischemia–reperfusion injury, all of which promote reactive oxygen species generation and cellular damage [46]. Oxidative stress contributes to tubular apoptosis, endothelial dysfunction, and mitochondrial injury, thereby accelerating nephron loss in structurally vulnerable kidneys.

Experimental studies suggest that antioxidant supplementation can attenuate oxidative damage and preserve renal structure in models of perinatal stress. Interventions including vitamin E, vitamin C, selenium, and pharmacologic redox modulators have demonstrated reductions in lipid peroxidation and inflammatory signaling, with associated improvements in renal histology [11,45]. However, translation into routine clinical practice has been limited by inconsistent human data and concerns regarding dosing, timing, and long-term safety.

Adjunctive perinatal strategies aimed at minimizing oxidative exposure may also indirectly protect renal development. Respiratory approaches that reduce oxygen toxicity and inflammatory lung injury have been associated with broader reductions in systemic oxidative stress and may confer downstream renal benefits [47]. Targeted antioxidant therapy, when combined with optimized respiratory and nutritional management, may represent a multifaceted approach to reducing oxidative burden during critical windows of renal vulnerability. Future research should focus on identifying biomarkers that reliably reflect renal oxidative stress in vivo and on developing targeted agents capable of modulating redox signaling without disrupting physiologic developmental processes.

### 6.3. Gut Microbiota and the Gut–Kidney Axis

Increasing evidence implicates the gut microbiome as a mediator of renal programming following preterm birth. The preterm gut microbiota is characterized by delayed colonization, reduced microbial diversity and enrichment of proinflammatory taxa, patterns that may persist beyond infancy and influence systemic immune and metabolic signaling [48]. Microbial-derived metabolites, including uremic toxins and short-chain fatty acids, can modulate endothelial function, inflammatory pathways, and renal perfusion, establishing bidirectional interactions between the gut and the kidney.

Host–microbiome interactions in preterm infants may therefore shape long-term susceptibility to kidney injury through immune priming and metabolic reprogramming [49]. Experimental studies suggest that modulation of the microbiota using probiotics, prebiotics, and synbiotics can attenuate systemic inflammation and reduce renal injury in models of CKD [50]. These interventions may influence gut barrier integrity, reduce endotoxemia, and alter production of nephrotoxic metabolites.

Human data in preterm populations remain limited, whereas optimal microbial targets and intervention windows have not been clearly defined. Nonetheless, the gut–kidney axis represents an attractive therapeutic frontier, particularly because microbiome-based interventions may be low risk and potentially applicable during early life. Integration of microbiome profiling with longitudinal renal outcome studies may enable identification of microbial signatures associated with renal resilience or vulnerability after prematurity.

### 6.4. Regenerative, Metabolic and Anti-Inflammatory Pathways

Beyond hemodynamic and oxidative mechanisms, emerging research is exploring strategies aimed at preserving nephron progenitor cell populations, enhancing mitochondrial resilience, and suppressing maladaptive inflammatory cascades. Experimental models suggest that inflammatory cytokine signaling during late nephrogenesis may impair nephron progenitor survival and promote fibrotic remodeling, linking perinatal inflammation to long-term nephron deficit [10].

Therapeutic approaches targeting metabolic reprogramming represent another promising avenue. Mitochondrial dysfunction and disturbed energy metabolism have been implicated in progressive nephron injury, whereas therapies that promote mitochondrial biogenesis or attenuate oxidative phosphorylation stress may enhance cellular resilience in structurally compromised kidneys [45]. Similarly, modulation of cellular senescence pathways and autophagy may influence long-term nephron survival, although these approaches remain largely preclinical.

Regenerative strategies, including stem cell-based therapies and growth factor modulation, aim to enhance nephron repair or replace injured renal tissue. While complete nephron regeneration in humans remains unlikely, paracrine effects of mesenchymal stem cells (MSCs) have shown promise in reducing inflammation and fibrosis in experimental kidney injury models. Translation to populations born preterm would require careful evaluation of safety, ethical considerations, and long-term oncogenic risk.

In this context, beyond traditional RAAS blockade, emerging pharmacological interventions and regenerative strategies offer new hope for mitigating the long-term renal consequences of prematurity. Sodium–glucose cotransporter 2 (SGLT2) inhibitors, originally developed to manage diabetes, have demonstrated potent nephroprotective effects by reducing glomerular hyperfiltration and intra-glomerular pressure, key pathophysiological features of the low nephron endowment seen in individuals born preterm [51,52]. Furthermore, the field of regenerative medicine is rapidly advancing, with recent studies on MSC-derived exosomes and kidney organoids offering a potential platform for reprogramming disrupted nephrogenesis. These bio-engineered models not only allow for a deeper understanding of the molecular pathways affected by preterm birth but also serve as vehicles for targeted delivery of epigenetic modulators that could reverse the developmental ‘hits’ incurred during the neonatal period [53,54].

### 6.5. Translational Challenges and Health-System Integration

Despite growing mechanistic insight, major challenges remain in translating experimental therapies into effective clinical interventions. Prematurity-related kidney disease is characterized by long latency, heterogeneous trajectories, and frequent absence of early symptoms, complicating trial design and outcome measurement. Identification of intermediate surrogate markers that accurately reflect nephron reserve and future CKD risk is therefore essential for evaluating preventive strategies.

Primary care systems also play a critical role in implementing emerging knowledge. Contemporary guidelines emphasize early identification of at-risk individuals, integration of renal risk assessment into routine chronic disease screening, and timely referral to specialist care when abnormalities are detected [55]. Embedding prematurity history into electronic health records and risk stratification tools may facilitate earlier recognition of vulnerable patients.

From a research perspective, multidisciplinary approaches integrating nephrology, neonatology, developmental biology, metabolomics, and microbiome science will be required to fully elucidate causal pathways and therapeutic windows. Longitudinal cohort studies with deep phenotyping, combined with pragmatic interventional trials, are likely to provide the strongest evidence base for future preventive strategies.

### 6.6. Toward Precision Prevention in Populations Born Preterm

Ultimately, the goal of emerging therapeutic research is to enable precision prevention, whereby interventions are tailored to individual risk profiles defined by gestational age, neonatal exposures, genetic susceptibility, and postnatal environmental factors. Advances in biomarker discovery, imaging technologies, and systems biology may allow earlier detection of maladaptive trajectories before irreversible nephron loss occurs.

Integration of predictive modeling with individualized follow-up schedules may permit targeted deployment of pharmacologic, nutritional, microbiome-based, and lifestyle interventions, maximizing benefit while minimizing unnecessary treatment. Such an approach aligns with the broader shift toward life-course medicine and offers a realistic framework for reducing the long-term burden of CKD attributable to preterm birth.

## 7. Conclusions

Preterm birth represents a major, non-modifiable determinant of reduced nephron endowment and lifelong renal vulnerability. The available epidemiologic and mechanistic evidence supports the interpretation that interrupted nephrogenesis establishes a developmentally reduced renal reserve that interacts with cumulative postnatal stressors across the life course. The combination of incomplete nephrogenesis, compensatory hyperfiltration, and cumulative postnatal insults therefore defines a trajectory that predisposes individuals born preterm to CKD, hypertension, and early functional decline. Early recognition of this susceptibility is not merely descriptive but clinically actionable, as timely surveillance and targeted interventions may mitigate long-term risk. A structured, life-course approach to monitoring and management, spanning infancy, childhood, adolescence, and adulthood, is critical for preserving renal health in this expanding population of preterm survivors.

Recognition of prematurity as an independent renal risk factor should translate into systematic clinical practice adjustments. Structured surveillance strategies incorporating blood pressure monitoring, assessment of albuminuria, and eGFR using both creatinine and cystatin C ought to be integrated into longitudinal follow-up frameworks from childhood through adulthood. Early detection of subclinical abnormalities provides a crucial window for intervention, including lifestyle modification and pharmacologic nephroprotection, which may help slow disease progression and preserve renal reserve.

As global survival of preterm infants increases, prematurity-related kidney disease is likely to contribute substantially to the future burden of CKD. Addressing this challenge requires coordinated care across neonatology, pediatrics, primary care, and adult nephrology to ensure continuity of follow-up across the life course. Embedding prematurity-specific renal risk awareness into standardized follow-up pathways represents a pragmatic and potentially high-impact strategy for reducing long-term CKD morbidity at the population level.

## 8. Future Directions

While this comprehensive review integrates epidemiologic, experimental and translational data to provide a life-course perspective on renal vulnerability after preterm birth, certain limitations should be acknowledged. As a narrative synthesis, it does not apply formal systematic review methodology or quantitative meta-analytic techniques, and heterogeneity across cohort definitions, gestational age categories, and outcome measures may limit direct comparability between studies. In addition, several mechanistic interpretations are informed by experimental models and require further validation in longitudinal human cohorts. Addressing these gaps through prospective studies incorporating imaging-based nephron estimation, biomarker validation, and risk-stratified follow-up frameworks will be essential to refine early identification strategies and strengthen the evidence base for targeted nephroprotection.

Future research should prioritize the development and validation of sensitive biomarkers capable of detecting early renal stress before irreversible nephron loss occurs. Promising candidates include NGAL, KIM-1, and longitudinal trajectories of cystatin C, which may provide greater sensitivity than serum creatinine in detecting subtle functional impairment [42,44]. Biomarker panels integrating inflammatory, metabolic, and tubular injury markers may further improve early risk stratification and enable identification of vulnerable individuals prior to clinical decline [31].

Advances in imaging technologies also offer opportunities for improved assessment of kidney structure and microvascular integrity. Functional MRI techniques and diffusion-weighted imaging, in combination with renal elastography, may enable non-invasive evaluation of nephron density, perfusion and tissue stiffness, offering potential surrogate markers of nephron reserve [43]. Integration of imaging data with biomarker profiles could enhance individualized prediction of long-term renal outcomes and improve longitudinal surveillance strategies [13,14].

Greater emphasis should also be placed on understanding epigenetic and molecular programming mechanisms linking prematurity to lifelong kidney vulnerability. Identification of epigenetic signatures and transcriptomic pathways associated with maladaptive renal development may reveal novel therapeutic targets and inform the timing of interventions during critical developmental windows [45]. Such approaches may ultimately allow modification of disease trajectories rather than solely delaying progression through downstream renoprotective therapies [9,10].

Finally, well-designed interventional trials are needed to evaluate strategies aimed at preventing or attenuating kidney injury both during neonatal intensive care and throughout later life. These should include trials of nephroprotective pharmacologic agents, optimized nutritional strategies, microbiome-targeted interventions, and long-term lifestyle programs tailored to populations born preterm [50]. The overarching aim is to advance toward precision nephroprotection, in which preventive and therapeutic strategies are individualized according to developmental history, genetic susceptibility, and postnatal exposures, thereby improving renal outcomes across the lifespan of preterm survivors.

## Figures and Tables

**Table 1:** Biomarkers for early detection of kidney injury and functional impairment in preterm-born populations.

Biomarker	Biological Source	Pathophysiological Significance	Advantages in Preterm Population
Cystatin C	Serum	Reflects glomerular filtration rate independent of tubular secretion	Independent of muscle mass, sex, and body size; minimal placental transfer; more accurate than creatinine in neonates and children
Neutrophil gelatinase-associated lipocalin (NGAL)	Urine/Serum	Marker of acute tubular epithelial injury and inflammatory activation	Enables early detection of structural kidney injury before creatinine rise; distinguishes structural from functional acute kidney injury (AKI)
Kidney injury molecule-1 (KIM-1)	Urine	Specific indicator of proximal tubular cell injury and regeneration	Highly sensitive to drug-induced nephrotoxicity (e.g., aminoglycosides, vancomycin) in the neonatal intensive care unit setting
β2-microglobulin	Urine	Reflects proximal tubular reabsorption capacity and tubular maturity	Useful marker of renal maturation and tubular dysfunction in very low birth weight infants
Interleukin-18 (IL-18)	Urine	Pro-inflammatory cytokine released from injured proximal tubular cells	Detects inflammatory tubular injury early; associated with ischemic and septic AKI in neonates

## Data Availability

The data supporting the reported results are from previously published studies. A list of all included studies is provided in the manuscript’s reference section. The original data presented in the study are openly available in all the academic databases mentioned in the method section.

## Abbreviations

The following abbreviations are used in this manuscript

CKD	Chronic Kidney Disease
AKI	Acute Kidney Injury
eGFR	estimated Glomerular Filtration Rate
DOHaD	Developmental Origins of Health and Disease
PDA	Patent Ductus Arteriosus
NSAID	Non-Steroidal Anti-Inflammatory Drugs
TGF-β	Transforming Growth Factor-β
NICU	Neonatal Intensive Care Unit
sCr	serum Creatinine
NGAL	Neutrophil Gelatinase-Associated Lipocalin
KIM-1	Kidney Injury Molecule-1
RAAS	Renin–Angiotensin–Aldosterone System
VEGF	Vascular Endothelial Growth Factor
SGLT2	Sodium–Glucose Cotransporter 2
MSC	Mesenchymal Stem Cell
VLBW	Very Low Birth Weight
ELBW	Extremely Low Birth Weight
MRI	Magnetic Resonance Imaging
ATP	Adenosine Triphosphate
CAKUT	Congenital Anomalies of the Kidney and Urinary Tract
